# A Case of Primary Submandibular Gland Oncocytic Carcinoma

**DOI:** 10.1155/2013/384238

**Published:** 2013-09-12

**Authors:** Kunihiko Tokashiki, Kiyoaki Tsukahara, Ray Motohashi, Kazuhiro Nakamura, Mamoru Suzuki

**Affiliations:** Otorhinolaryngology, Head & Neck Surgery, Tokyo Medical University Hachioji Medical Center, 403 La Conte Shinjuku, Nishi-Shinjuku 5-8-6, Shinjuku-ku, Tokyo 160-0023, Japan

## Abstract

Primary submandibular gland oncocytic carcinoma is a rare pathology, with only 10 cases being reported to date. We encountered a case of primary submandibular gland oncocytic carcinoma and report it herein. The patient was a 69-year-old man who came to our hospital with right submandibular cancer as the main complaint. Based on the results of computed tomography and magnetic resonance imaging, submandibular gland tumor was diagnosed. Preoperative cytodiagnosis suggested class III oncocytic carcinoma. Resection of the right submandibular tumor was performed along with right neck dissection. Postoperative histopathological diagnosis was oncocytic carcinoma. As of 3 years following surgery, no recurrence has been identified.

## 1. Introduction

Oncocytes are epithelial cells with cytoplasm rich in eosinophilic granules. Oncocytoma is a cancerous proliferation of oncocytes that accounts for only 1% of all salivary gland tumors [1]. Oncocytic carcinoma is an even rarer malignancy that starts in the parotid glands in almost all cases. Only 10 cases of primary submandibular gland oncocytic carcinoma have been reported to date [2–10] (Table 1). Herein, we report a case of primary submandibular gland oncocytic carcinoma.

## 2. Case Presentation

**Figure 1:**
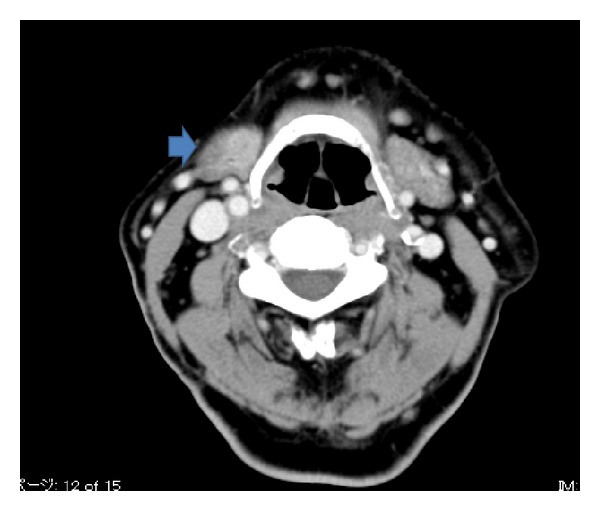
Neck CT showing a 20 mm × 10 mm nodular shadow with slight enhancement in the right submandibular area.

**Figure 2:**
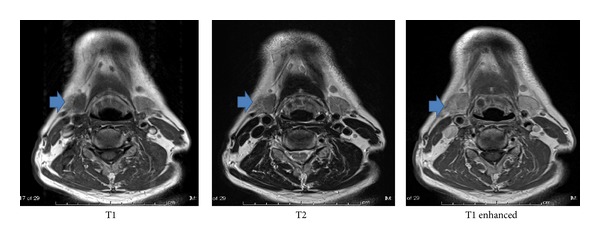
Neck MRI showing 20 mm × 10 mm tumor with an indistinct boundary and slight enhancement in the right submandibular gland. Signal intensities are low on both T1- and T2-weighted images.

**Figure 3:**
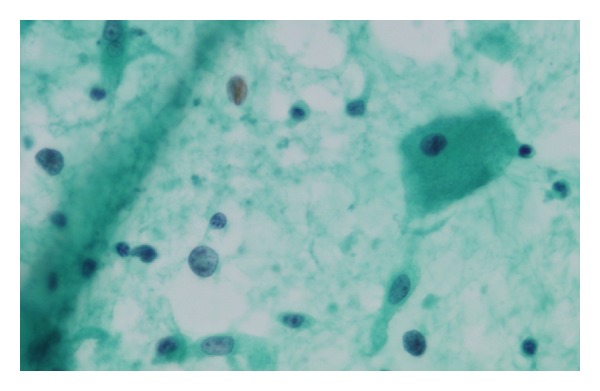
Fine-needle aspiration showing the eosinophilic cytoplasm of duct epithelial cells and the presence of naked nuclei with prominent nucleoli.

The patient was a 69-year-old man who was examined for the main complaint of right submandibular cancer. The medical and family histories of the patient were unremarkable. Initial examination revealed a 15 mm × 10 mm hard, elastic tumor showing no mobility and an irregular margin. On computed tomography (CT) of the neck, a 20 mm × 10 mm nodular shadow with slight enhancement was observed in the right submandibular area (Figure 1). No clear indication of metastasis to cervical lymph nodes was apparent. No obvious distant metastases were observed on chest CT. Neck magnetic resonance imaging (MRI) revealed a 20 mm × 10 mm tumor with indistinct boundary and slight enhancement in the right submandibular gland, showing signal hypointensity on both T1- and T2-weighted imaging (Figure 2). Fine-needle aspiration (FNA) biopsy at this location indicated a class III lesion. The eosinophilic cytoplasm of duct epithelial cells and the presence of naked nuclei with prominent nucleoli made it difficult to rule out malignancy (Figure 3).

**Figure 4:**
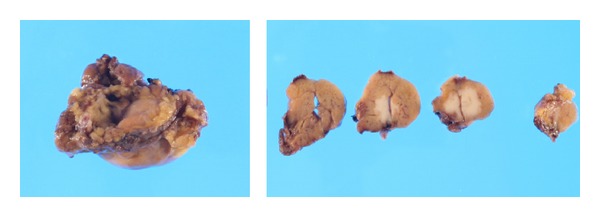
The 20 mm × 10 mm tumor. The smooth surface of the removed tumor is light brown in color with a solid appearance.

**Figure 5:**
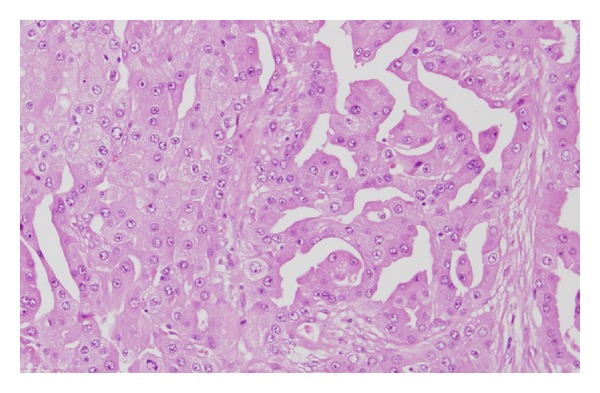
Hematoxylin-eosin staining showing a tumor with extensive cytoplasm containing eosinophilic granules in an irregular papillary ring structure. Infiltration of the surrounding salivary gland tissue was also observed. No infiltration of lymph ducts, blood vessels, or nerve peripheries was evident, and margins were negative.

**Figure 6:**
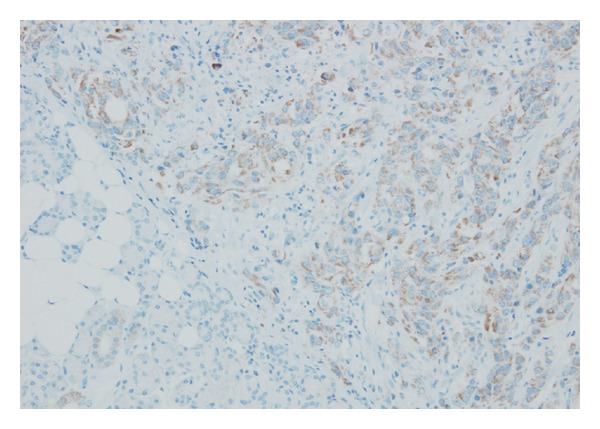
Antimitochondrial antibody staining showing more tissue with fine blue-stained granules than normal tissue.

In view of these findings, we explained the possibility of malignancy to the patient and after gaining informed consent resected the right submandibular gland tumor and conducted right neck dissection. Although no obvious metastasis to lymph nodes was identified, we removed the lymph nodes and submandibular gland together. No adhesion to the surrounding area was apparent. We preserved cranial nerves VII, IX, X, XI, and XII and the cervical nerves. The smooth surface of the removed tumor was light brown in color and solid in appearance (Figure 4). No particular complications were encountered postoperatively, and the patient was discharged 7 days later. On histopathological examination, hematoxylin and eosin staining revealed a tumor with extensive cytoplasm containing eosinophilic granules in an irregular papillary ring structure. Infiltration of the surrounding salivary gland tissue was also observed. No infiltration of lymph ducts, blood vessels, or nerve peripheries was seen and the stump of the extracted tumor was negative (Figure 5). On antimitochondrial antibody staining, more tissue with fine blue-stained granules than normal tissue was seen (Figure 6). Based on these results, oncocytic carcinoma was diagnosed.

As histopathology had revealed no infiltration of lymph ducts, blood vessels, or nerve peripheries and the surgical margins were negative, no postoperative treatment was conducted. The patient has been followed up on an outpatient basis, with no recurrence or metastases being identified as of the time of writing, 3 years postoperatively.

## 3. Discussion

Pathologically, distinguishing oncocytic carcinoma from oncocytoma is extremely difficult. Some of the diagnosis criteria for degree of malignancy are (1) distant metastasis; (2) lymph node metastasis; (3) nerve or blood vessel infiltration; and (4) atypical cancer cells and mitosis [11, 12]. In the present case, preoperative FNA biopsy showed that the degree of atypicality was low, but malignancy was difficult to rule out and the tumor was determined to be class III in the Papanicolaou classification. Given the possibility of malignancy, surgery was performed. In view of the irregular, papillary ring structure infiltrating into neighboring salivary gland tissue seen in reference postoperative histopathological specimens, low-grade oncocytic carcinoma was diagnosed.

Diagnosis made on the basis of FNA may be inadequate. In addition, pathological diagnosis of this tumor is very difficult. We consider that surgical resection is warranted if the possibility of malignancy remains. In all such cases to date, surgery has been the first-choice intervention (Table 1). Although radiotherapy or chemotherapy as well as adjunctive therapy may be conducted, the usefulness of these methods has not been established. However, there is a tendency to also conduct radiotherapy in cases with metastasis to lymph nodes or high-grade malignancy.

In the present case, as there was no evidence of lymph node metastasis or infiltration of lymph ducts or blood vessels and surgical margins were negative on histopathological examination, the grade of malignancy was determined to be low, and no additional therapy was conducted.

The prognosis varies with the degree of malignancy. Also, there are cases of progression in the relatively short term and those in which recurrence appears after 10 years. A followup must therefore be matched to the patient. In the present case, no recurrence or metastasis has been encountered as of 3 years postoperatively, and a followup will be continued in the future.

We have reported a case of primary submandibular gland oncocytic carcinoma. While no definitive diagnosis could be made from preoperative cytodiagnosis, a definitive diagnosis was reached from postoperative histopathology. Treatment involved complete submandibular gland removal and right neck dissection, and the patient has remained free of recurrence and metastasis in the 3 years since surgery.

## Figures and Tables

**Table 1:** Reports of oncocytic carcinoma in submandibular glands.

Author	Year	Sex	Age (years)	Treatment	Clinical course
Goode et al. [2]	1988	M	68	Ope	Alive, 16 years
Brandwein and Huvous [3]	1991	M	62	Unknown	Alive, 6 months
Ziegler et al. [4]	1992	F	56	Ope + Ct + Rt	Alive, 9 months
Wu and Silvernagel [5]	1998	F	77	Ope	Alive, 6 months
Nakada et al. [6]	1998	M	69	Ope + Rt	Alive, 1.5 year
Muramatsu et al. [7]	2002	M	82	Ope	Died after 1 year
Wischerath et al. [8]	2002	M	59	Ope + Rt	Unknown
Mizutari et al. [9]	2005	M	55	Ope + Rt	Alive, 1.5 year
Lee and Chang [10]	2010	M	51	Ope	Unknown
